# Analytical network process based optimum cluster head selection in wireless sensor network

**DOI:** 10.1371/journal.pone.0180848

**Published:** 2017-07-18

**Authors:** Haleem Farman, Huma Javed, Bilal Jan, Jamil Ahmad, Shaukat Ali, Falak Naz Khalil, Murad Khan

**Affiliations:** 1 Department of Computer Science, University of Peshawar, Peshawar, Pakistan; 2 Department of Computer Science and IT, Sarhad University of Science and IT, Peshawar, Pakistan; 3 Department of Control and Computer Engineering, Politecnico di Torino, Turin, Italy; 4 College of Electronics and Information Engineering, Sejong University, Seoul, South Korea; Nankai University, CHINA

## Abstract

Wireless Sensor Networks (WSNs) are becoming ubiquitous in everyday life due to their applications in weather forecasting, surveillance, implantable sensors for health monitoring and other plethora of applications. WSN is equipped with hundreds and thousands of small sensor nodes. As the size of a sensor node decreases, critical issues such as limited energy, computation time and limited memory become even more highlighted. In such a case, network lifetime mainly depends on efficient use of available resources. Organizing nearby nodes into clusters make it convenient to efficiently manage each cluster as well as the overall network. In this paper, we extend our previous work of grid-based hybrid network deployment approach, in which merge and split technique has been proposed to construct network topology. Constructing topology through our proposed technique, in this paper we have used analytical network process (ANP) model for cluster head selection in WSN. Five distinct parameters: distance from nodes *(DistNode)*, residual energy level *(REL)*, distance from centroid (*DistCent*), number of times the node has been selected as cluster head *(TCH)* and merged node (*MN*) are considered for CH selection. The problem of CH selection based on these parameters is tackled as a multi criteria decision system, for which ANP method is used for optimum cluster head selection. Main contribution of this work is to check the applicability of ANP model for cluster head selection in WSN. In addition, sensitivity analysis is carried out to check the stability of alternatives (available candidate nodes) and their ranking for different scenarios. The simulation results show that the proposed method outperforms existing energy efficient clustering protocols in terms of optimum CH selection and minimizing CH reselection process that results in extending overall network lifetime. This paper analyzes that ANP method used for CH selection with better understanding of the dependencies of different components involved in the evaluation process.

## 1. Introduction

Wireless sensors are widely used for health and environmental monitoring, smart homes, smart transportation, and rescue operations [1]. Wireless sensor nodes are usually operated on batteries often in unattended environments where batteries cannot be recharged and battery drainage can cause network disconnection. Sensor nodes consume most of their energy during communication with other nodes [2]. It is highly desirable to optimize communications which can lead to effective and efficient usage of limited resources, thereby enhancing network lifetime.

**Fig 1:**
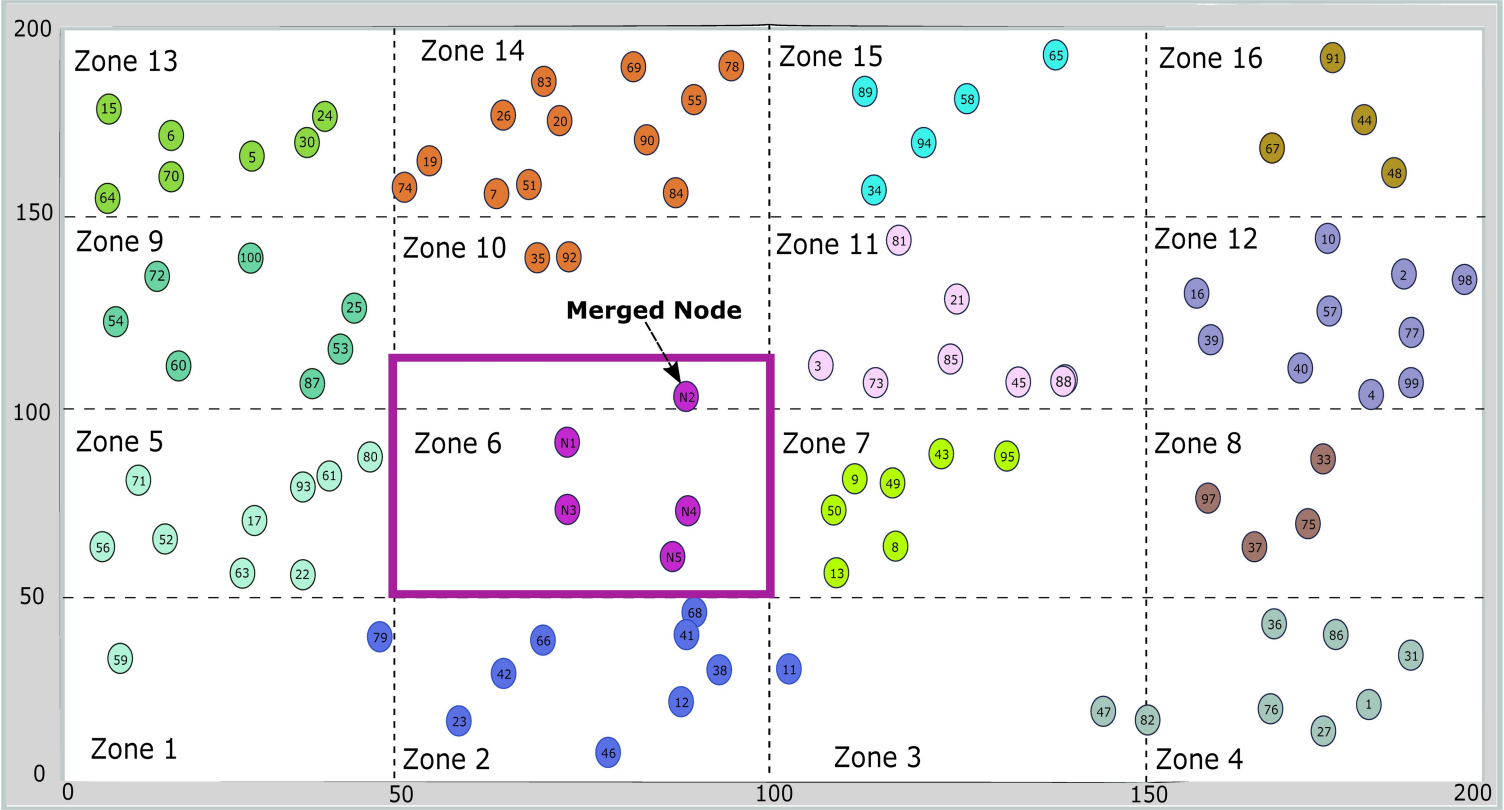
Grid-based hybrid network deployment.

Over the years clustering has been used for establishing network in a hierarchical manner. An energy efficient and robust way of transmission can be achieved through clustering approach in which nodes are grouped and organized in to small clusters [3]. Each cluster has a cluster head responsible for forwarding aggregated data from member nodes to the base station (BS) directly or through a sequence of CHs. Clustering provides many advantages over flat architecture such as reducing inter-node communication, network scalability, bandwidth management and allowing nodes to sleep for a period of time resulting in energy savings [3]. CH selection is an important part of any cluster-based approach that can directly affect network performance. CH selection based on single criteria (e.g. residual energy) can lead to poor performance because the selected CH may not be a good choice [3]. In addition to residual energy, other parameters such as distance from other nodes and cluster centroid etc. should also be considered for optimum CH selection. In our previous work, grid based hybrid clustering is used where the sensor network is partitioned into multiple virtual grids as shown in Fig 1. Nodes are grouped into corresponding clusters (zones) based on their location determined by the coordinates. The role of cluster head is rotated in order to maximize the network lifetime. In current work, we have extended cluster head selection criteria to five distinct parameters so that the most effective node can be selected as a cluster head using ANP approach. The parameters are shown in Table 1 which are REL, DistNodes, DistCent, TCH and MN.

**Table 1:** Criteria parameters.

S.No	Terminology	Description
01	REL	Residual Energy Level of a node
02	DistNodes	Distance of a node from its neighboring nodes in the current zone
03	DistCent	Distance of a node from the center of zone
04	TCH	Number of times a node has been selected as Cluster Head
05	MN	Node that has been in-grouped from the neighboring low density zone.

In this paper, a multi-decision criteria tool Analytical Network Process (ANP) is used to select the most suitable node as a CH among available nodes. The multi criteria decision tool ANP was introduced to solve dependency problems for different elements and the feedback between network layers which was missing in its previous version Analytical Hierarchy Process (AHP) [4]. We have used ANP because it can deal with the interdependencies and feedback relationship between elements and clusters. The objective of this work is to use ANP as a multi-criteria decision tool for optimum cluster head selection in WSN. The proposed work is compared with existing state of the art protocols to evaluate performance in terms of network lifetime.

Rest of the paper is organized as follows. Section 2 presents related work. The ANP method is briefly explained in section 3. In section 4, the proposed work of cluster head selection using ANP is described briefly. Sensitivity analysis for different scenarios is given in section 5. The section 6 briefly presents cluster head reselection process. The results and discussions are presented in section 7. The paper is concluded in section 8 with future directions.

## 2. Related work

In literature authors have addressed the issue of energy efficiency through different approaches such as classical clustering, chain-based clustering and grid based clustering. The aim of all approaches is to efficiently use available resources to prolong network life time. Few of them are discussed here.

Low energy adaptive clustering hierarchy (LEACH) [5] was the first attempted cluster based routing protocol for wireless sensor network proposed by W. Heinzelman. It works in a distributed manner and has no centralized control. Nodes forward their data to cluster head which is selected randomly with a certain probability in each round. Nodes selects a random number between 0 and 1 and if the number is less than a specific threshold then they will elect themselves as CH for that specific round. To avoid early drainage of battery CH selection is rotated. Node once selected as CH, cannot be considered again in that round. One of the limitation of LEACH is no control over CH, as far as the nodes are generating random numbers less than specific threshold, they will elect themselves as CH. Secondly, due to probabilistic approach, LEACH doesn’t consider residual energy in to account while selecting CH and finally it is not suitable for large scale networks.

LEACH limitations were addressed by different authors as number of CH variability was addressed in stepwise adaptive clustering hierarchy (SWATCH) [6]. To keep the CH count in the optimal range, setup phase is divided in to two phases: Initial selection phase and add-on selection phase. The problem of selection of low energy nodes was addressed by LEACH with deterministic cluster head selection (LEACH-DCHS) [7]and advance LEACH [8]. Energy efficient clustering scheme (EECS) [9], enhanced centralized LEACH (EC-LEACH) [10] and hybrid energy efficient distributed (HEED) [11] solved the problem of non-uniform distribution of CH. In LEACH-MAC [1] the problem of randomness in CH selection was controlled and to make the CH count stable.

In power efficient gathering in sensor information systems (PEGASIS) [12] a chain of nodes is constructed using greedy algorithm. Each node receives a single packet from its neighboring node and will apply data fusion along with its own data and will forward to the next neighboring node in the chain. All the data collected from chain will be forwarded by the chain leader to the BS. Energy efficient chain based (EECB) [13] improves PEGASIS by avoiding the elongated links on chain to uniformly distribute the load by applying the distance threshold. On the basis of a function that is remaining residual energy and distance between node and BS, leader is selected to forward data to the BS. Energy efficient chain based network (ECBSN) [14] is based on PEGASIS, in which two layers, low and high, are constructed. It has multiple chains and each chain has its own leader. Multiple low layer chain leaders form a high layer chain. Leader having shortest distance to the BS will be selected as high layer chain leader.

In grid based data dissemination (GBDD) [15], BS divides the whole network into virtual grids. Node that is interested in communication is set as cross point for that grid and is used as a reference point for other nodes and for grid creation. In CBDAS [16], the whole network is divided into cells and each cell has a cell head that is responsible for collecting and forwarding of data. All cell heads are linked to form a chain and the one having high residual energy is selected as cycle head to communicate with BS. In grid-based hybrid network deployment (GHND) [17], merge and split technique was used to evenly distribute the load across the network. If the number of nodes are low from lower threshold, they will be merged with neighboring zone depending on density and distance. Number of nodes if exceeds from upper threshold, that zone will be split in to sub-zones.

Multi criteria decision making (MCDM) is used, where one component among multiple components is to be selected on the basis of some criteria [18]. MCDM is used in several fields of science such as natural resource management [19], software engineering [20], complex networks [21] and many more. Different techniques are used in literature for selection to get the desire goal such as Analytical Hierarchy Process (AHP), ANP and many others. In [22], a centralized cluster head selection approach using AHP is proposed. Three factors, mobility, energy and distance to the center of the cluster were considered. Authors claim that AHP performs better than LEACH. The ANP has also been used as a tool for MCDM such as in [23], suitable waste water treatment (WWT) technology was selected using ANP approach. In [24], ANP was used along with risk priority index to model risks in megaprojects. Shah Nazir et. al [25] on the basis of quality criteria used ANP for software component selection. We believe that ANP method can also be applied to analyze the structure and dynamic behavior of complex systems. Gradually complex systems in the real world are becoming dependent on each other due to which interdependent networks has become an active part in network science [26]. ANP has been widely used as a multi criteria decision making tool. Complex systems in the real world are dependent on each other,

## 3. Analytical network process

**Fig 2:**
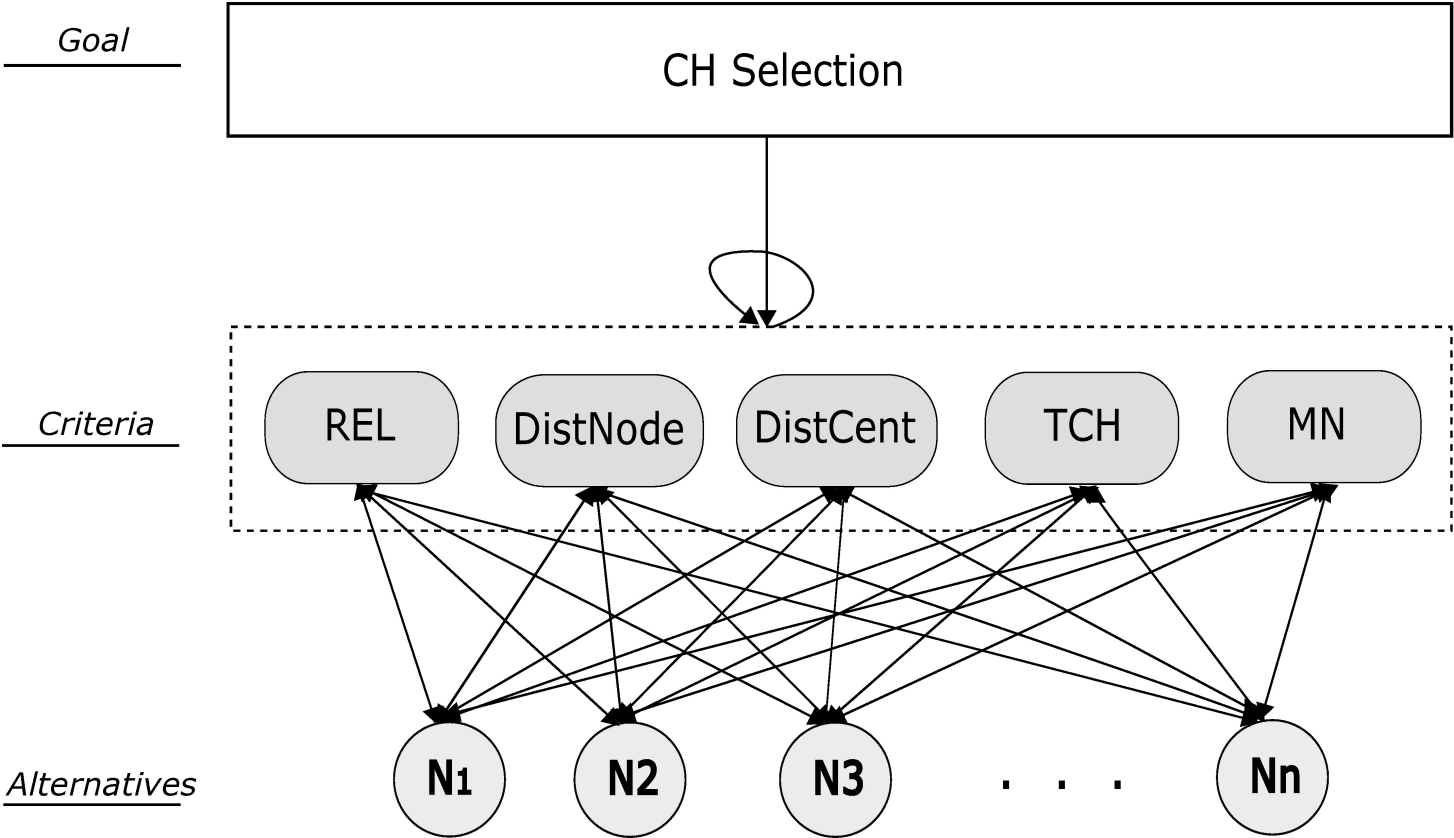
ANP model for CH selection.

ANP was developed by Saaty [27] on the basis of Analytical Hierarchy Process (AHP). ANP can deal with the qualitative and quantitative information of the network. In addition it also handles interaction and feedback relationships between the criteria/sub-criteria and alternatives. The ANP model has been used as the multi-criteria decision tool for different purposes such as, project selection, component selection e. The generalized steps [27, 28] of ANP are discussed as:

1. The main problem is divided in to sub problems by clearly identifying the goal, criteria/sub-criteria and alternatives as shown in Fig 2. Goal is what we want to achieve, criteria is the set of parameters on which decision depends and alternatives are the elements upon which decision has to be made.
2. Criteria and alternatives are scaled according to the qualitative scale of importance introduced by Saaty [27] and then it is converted to the quantitative scale range that is from 1 to 9 as shown in Table 2.
3. Pairwise comparison is done after scaling. Matrix of criteria is created by comparing the *ith* row with the *jth* column. If the *ith* row criteria is superior then it is denoted by *(i*, *j)* otherwise *(j*, *i)*. A score of 1 represents equal importance, whereas 9 represents extreme importance of one element over other.
4. Relative importance is calculated through Eigen values and Eigen vector of the comparison matrix. Weights of the criteria or sub-criteria are obtained as the elements are normalized.
5. In order to assure the judgments reliability, it is very important to ensure the consistency between the comparisons made. The Consistency Index (CI) and Consistency Ratio (CR) are defined by Saaty [29] and briefly explained in section 4.2.1. CR is the consistency ratio and RI is consistency index of the random reciprocal matrix generated from the quantitative 9-point scale [27]. The value of *CR* ≤ *0*.*1*is acceptable otherwise the pairwise comparison needs to be revised.
6. The local priority values (Eigen vectors) obtained from comparison matrix results in unweighted supermatrix. It is transformed into weighted supermatrix if summation of each column is 1 otherwise, there is interdependence between the clusters in a network. Weighted supermatrix is the outcome of unweighted supermatrix and cluster matrix.
7. The Limit matrix is obtained by raising the weighted supermatrix to the power of *2k* until it is converged to get more stable set of weights, where *k* is arbitrary large number. The final priorities of all elements in the network can be obtained by normalizing each block of the limit matrix. The best alternative should be selected having largest priority.
8. In the end a sensitivity analysis is performed to check the results stability and ranking of the alternatives provided by whole ANP process.

**Table 2:** Quantitative 9-point scale.

Scale	Description
1	Equal relative importance
2	Equally to moderately more important
3	Moderately more important
4	Moderately to strongly important
5	Strongly important
6	Strongly to very strongly more important
7	Very strongly more important
8	Very strongly to extremely more important
9	Extremely important (high priority)

## 4. Cluster head selection using ANP method

The performance of cluster-based WSN directly depends on CH, therefore it is very important to select the optimum node that will ensure efficient resource utilization, thereby improving network lifetime. In this paper, the topology for CH selection is based on grid-based hybrid network deployment (GHND) as in [17]. The constructed topology is shown in Fig 1, where the network is partitioned in multiple zones. Each zone has a CH responsible for data aggregation and forwarding. Cluster head plays a very important role in network stability and prolonging network life time, therefore it should be intelligently selected. The problem of selecting the best node as CH based on certain parameters and can be easily tackled as a multi criteria decision system. ANP has been widely used as a multi criteria decision tool, in which dependencies among elements and feedback are dealt with. After the network deployment, the base station (BS) will run the ANP based CH selection for all clusters. The steps involved in ANP model for cluster head selection are explained in detail as follows:

### 4.1 Problem formulation

**Fig 3:**
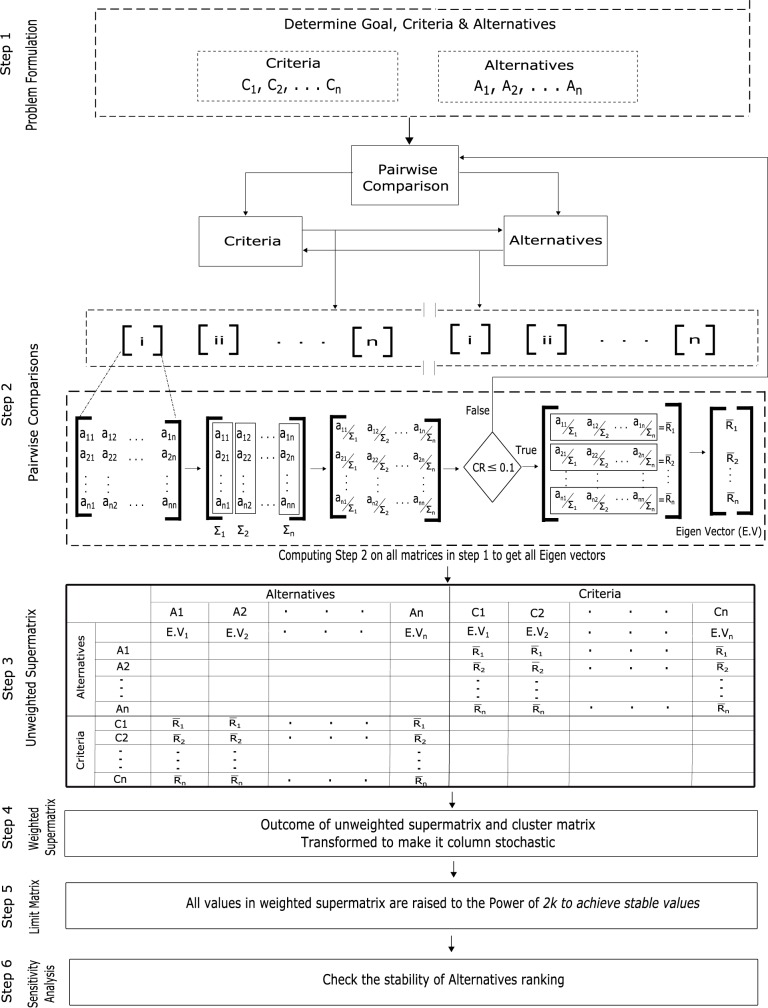
Steps involved in ANP model of CH selection.

The ANP model structures the given problem into goal, criteria and alternatives as shown in Fig 2. In this paper our goal is to select the best cluster head among the alternatives in a specific cluster. For example in Fig 1, cluster head is to be selected in the highlighted cluster. Parameters are REL, DistNode, DistCent, TCH and MN. Alternatives are sensor nodes available in the selected cluster. Criteria and alternatives are represented by two sets, ***C*** and ***A*** respectively. Relationship between criteria and alternatives is identified for constructing a network along with their dependencies. The whole process of ANP is initially applied for cluster head selection in a certain zone as shown in Fig 3. The whole process is repeated for all zones in the network.

$$\begin{array}{l} \mathrm{Set}\;1:\;C=\{REL,DistNode,DistCent,TCH,MN\}\\ \mathrm{Set}\;2:\;A=\{N1,N2,N3,N4,N5\} \end{array}$$

### 4.2 Pairwise comparison of elements

Pairwise comparison is the method of establishing the comparative significance of different elements (criteria and alternatives) with respect to a specific component in the network. The same is also used to judge quantitative impact of elements on the goal and is scaled according to Saaty 9-point scaling [27] shown in Table 2. A score of 1 represents equal importance, whereas 9 represents extreme importance of one element over other. These quantitative numerical values, representing personal judgments, must be carefully assigned to both criteria and alternatives set elements for drawing the pairwise comparisons. For example, checking the impact of criterion *DistNodes* on alternatives such as N_1_ and N_2_. As N_2_ is relatively closer to other nodes in the cluster than N_1_ (as shown in section 5.1, scenario 1)), therefore its quantitative importance is set to scale 3 (explained in section 4.2.1). Matrix of criteria is created by comparing the *ith* row with the *jth* column. If the *ith* row criteria is superior then it is denoted by *(i*, *j)* otherwise *(j*, *i)*. The relative weights of elements are represented in square matrix of order *n* which is shown as *a*_*ij*,_(*a* represents elements as shown in matrix (1)) where *i* and *j* represents row and column respectively. The diagonal elements having same importance are represented by 1 as shown in matrix (1).

$$\left[\begin{array}{cccccc} & a_{1} & a_{2} & a_{3} & a_{4} & a_{5}\\ a_{1} & 1 & & & & \\ a_{2} & & 1 & & & \\ a_{3} & & & 1 & & \\ a_{4} & & & & 1 & \\ a_{5} & & & & & 1 \end{array}\right]$$ (1)

#### 4.2.1 Alternatives comparison using criteria

For each criteria, all the elements of alternatives are pairwise compared with each other. Once all the alternatives are compared, we will get comparison matrix, its general form is shown in matrix (2), where C_1_ to C_n_ represents columns and R_1_ to R_n_ represents rows. Starting from *DistCent* as the first criteria element, all alternatives are compared with each other (represented by N_1_ to N_5_) according to the 9-point scale (as shown in Table 2). Entries above the diagonal values in matrix (3) is obtained from the pairwise comparison according to quantitative 9-point scale (Table 2), the entries below the diagonal values are generated automatically with the corresponding reciprocal values. Initially N_1_ is compared with N_2_, N_3_, N_4_ and N_5_ taking into account the *DistCent* parameter resulting in row 1 of matrix (3). In this row, N_1_ is of same importance to itself (represented by value 1), N_1_ is moderately more important than N_2_, N_3_ is strongly to very strongly more important than N_1_ (scaling value 8, here represented by reciprocal value) and nodes (N_4_ and N_5_) are equally to moderately more important than N_1_ (represented by scaling value of 2, here represented by reciprocal values).

$$\left[\begin{array}{cccccc} & C_{1} & C_{2} & C_{3} & \ldots & C_{n}\\ R_{1} & a_{11} & a_{12} & a_{13} & \ldots & a_{1n}\\ R_{2} & \frac{1}{a_{12}} & a_{22} & a_{23} & \ldots & a_{2n}\\ R_{3} & \frac{1}{a_{13}} & \frac{1}{a_{23}} & a_{33} & \ldots & a_{3n}\\ \vdots & \vdots & \vdots & \vdots & \ldots & \vdots \\ R_{n} & \frac{1}{a_{1n}} & \frac{1}{a_{2n}} & \frac{1}{a_{3n}} & \ldots & a_{nn} \end{array}\right]$$ (2)

$$\left[\begin{array}{cccccc} & N_{1} & N_{2} & N_{3} & N_{4} & N_{5}\\ N_{1} & 1 & 2 & \frac{1}{8} & \frac{1}{2} & \frac{1}{2}\\ N_{2} & \frac{1}{2} & 1 & \frac{1}{8} & 2 & \frac{1}{2}\\ N_{3} & 8 & 8 & 1 & 8 & 8\\ N_{4} & 2 & \frac{1}{2} & \frac{1}{8} & 1 & \frac{1}{2}\\ N_{5} & 2 & 2 & \frac{1}{8} & 2 & 1 \end{array}\right]$$ (3)

After pairwise comparison, all the elements of each column in matrix (3) are summed using Eqs (4) and (5). The result of this summation is shown in matrix (6). Eq (4) shows the process for C_1_ to C_n-1_ and Eq (5) is for last column (C_n_), where *Si* represents the respective column sum.

For C_1_ to C_n-1_

$$S_{i}=\sum_{j=1}^{k-1}a_{ji}+\sum_{j=k}^{n}\frac{1}{a_{ij}}\;\mathrm{where}\;k=2,3,4,\ldots ,n\;\mathrm{and}\;i=1,2,3,\ldots ,n\;\text{-}\;1$$ (4)

For C_n_

$$S_{n}=\sum_{j=1}^{k-1}a_{ji}\;\mathrm{where}\;k=n+1,\;i=n$$ (5)

$$\left[\begin{array}{cccccc} & N_{1} & N_{2} & N_{3} & N_{4} & N_{5}\\ N_{1} & 1.0 & 2.0 & 0.125 & 0.5 & 0.5\\ N_{2} & 0.5 & 1.0 & 0.125 & 2.0 & 0.5\\ N_{3} & 8.0 & 8.0 & 1.0 & 8.0 & 8.0\\ N_{4} & 2.0 & 0.5 & 0.125 & 1.0 & 0.5\\ N_{5} & 2.0 & 2.0 & 0.125 & 2.0 & 1.0\\ Total & 13.5 & 13.5 & 1.5 & 13.5 & 10.5 \end{array}\right]$$ (6)

Matrix (7) is the normalized form of matrix (6), in which each column individual value is divided by the sum of respective columns (*Si*).

$$\left[\begin{array}{cccccc} & N_{1} & N_{2} & N_{3} & N_{4} & N_{5}\\ N_{1} & \frac{1.0}{13.5}=0.074 & \frac{2.0}{13.5}=0.148 & \frac{0.125}{1.5}=0.083 & \frac{0.5}{13.5}=0.037 & \frac{0.5}{10.5}=0.047\\ N_{2} & \frac{0.5}{13.5}=0.037 & \frac{1.0}{13.5}=0.074 & \frac{0.125}{1.5}=0.083 & \frac{2.0}{13.5}=0.148 & \frac{0.5}{10.5}=0.047\\ N_{3} & \frac{8.0}{13.5}=0.592 & \frac{8.0}{13.5}=0.592 & \frac{1.0}{1.5}=0.666 & \frac{8.0}{13.5}=0.592 & \frac{8.0}{10.5}=0.762\\ N_{4} & \frac{2.0}{13.5}=0.148 & \frac{0.5}{13.5}=0.037 & \frac{0.125}{1.5}=0.083 & \frac{1.0}{13.5}=0.074 & \frac{0.5}{10.5}=0.047\\ N_{5} & \frac{2.0}{13.5}=0.148 & \frac{2.0}{13.5}=0.148 & \frac{0.125}{1.5}=0.083 & \frac{2.0}{13.5}=0.148 & \frac{1.0}{10.5}=0.095 \end{array}\right]$$ (7)

Each individual row is summed and their average is taken to get Eigen vector (EV). To check the reliability of pairwise comparisons (consistency check), the consistency metrics used are: Consistency Measure (CM), Consistency Index (CI) and Consistency Ratio (CR).

**Fig 4:**
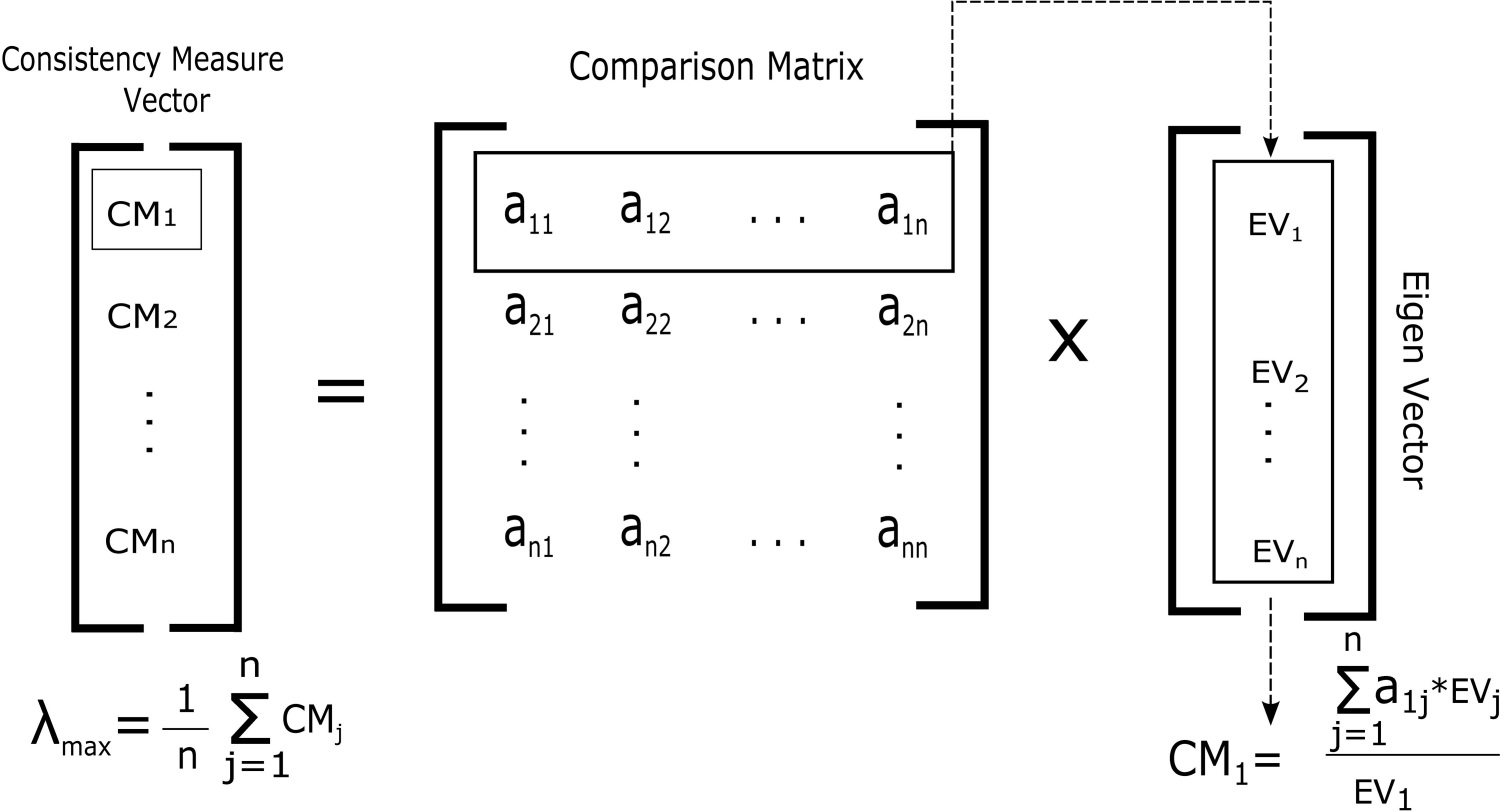
Consistency measure calculation.

*Consistency Measure*: Consistency Measure (CM) is the first step in making consistency analysis. The CM vector is input for consistency index and ratio (CI and CR) calculation. In order to find CM, matrix (6) is first row-wise multiplied with the Eigen vector as in matrix (12) and then divided by the corresponding element of the *EV* as shown in Fig 4. The general form for obtaining CM is given in Eq (8), where *R*_*j*_ is the corresponding row of comparison matrix, *EV* is the Eigen vector (priority vector) and *EV*_*j*_ represents the corresponding element in *EV*. The average of *CM* vector is *λ*_*max*._

$$CM_{j}=\frac{R_{j}\times EV}{EV_{i}}\;\mathrm{where}\;j=1,2,3,\ldots ,\;n$$ (8)

*Consistency Index*: According to Saaty [24], CI is the degree or deviation of consistency. To calculate CI for *DistCent* iteration, we need to put the values of *λ*_*max*_ *= 5*.*37* and *n* = 5 in Eq (9) results in *CI* = 0.092.

$$CI=\frac{\lambda_{\max}-n}{n-1}$$ (9)

**Fig 5:**
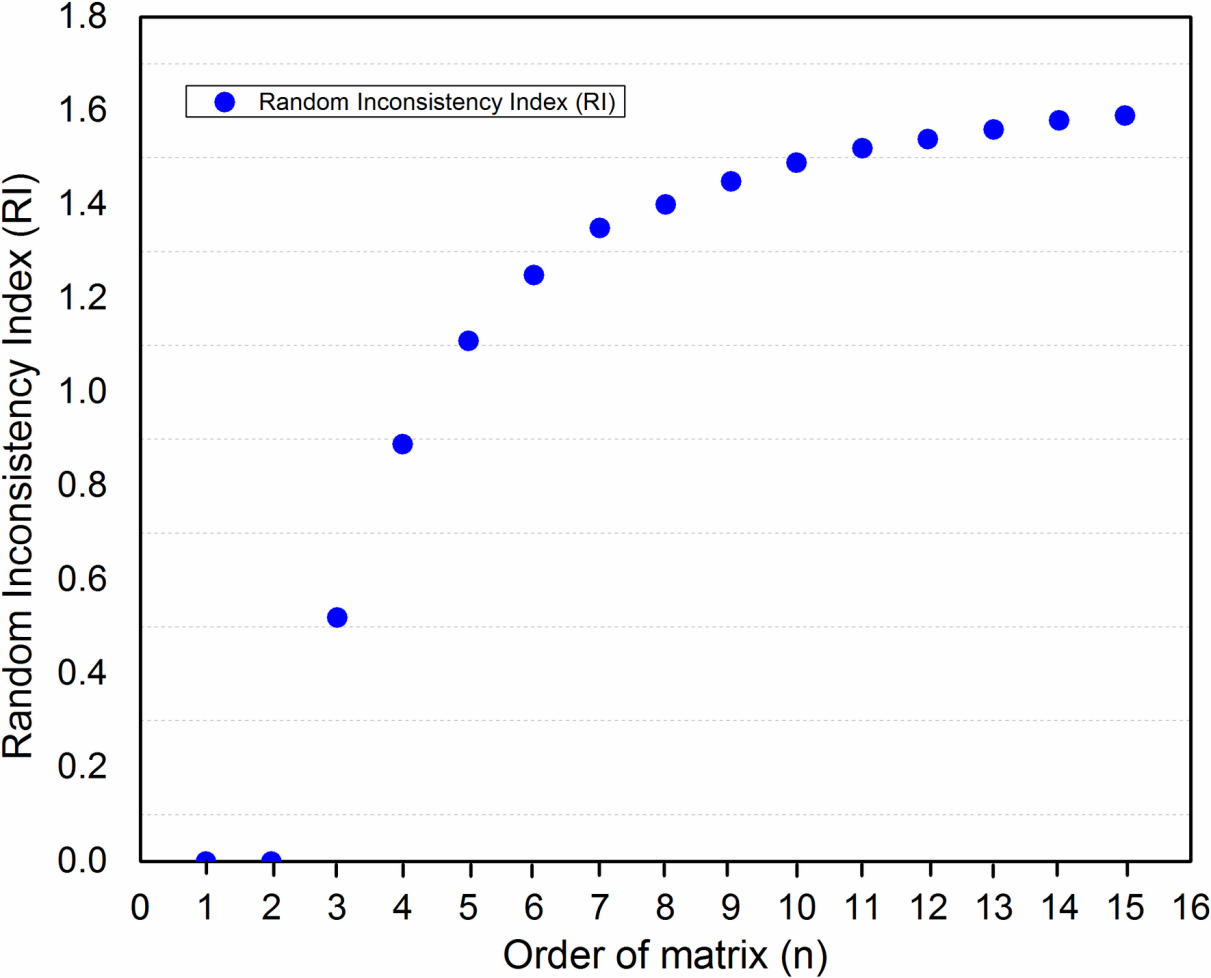
Random inconsistency.

*Consistency Ratio*: To check the reliability of pairwise comparisons, it is very important to ensure the consistency between the pairwise comparisons made. To get consistency ratio (CR), we need to calculate consistency index (CI) as shown in Eq (9) and *RI* (value of *RI* is drawn from Table 3 according to the order of matrix represented by *n*, in our case *n* = 5). *RI* is consistency index of the random reciprocal matrix generated from the quantitative 9-point scale. The value of *n* in Table 3 have been experimentally obtained as in [28]. For the order of matrix greater than 9, the values for RI are nearly leveled with negligible change as shown in Fig 5. However, in literature researchers have proposed the order of matrix greater than 9 to calculate RI [30]. Putting the values of *CI* (from Eq (9)) and *RI* (from Table 3) in Eq (10) results in *CR = 0*.*08* which means that inconsistency is 8% satisfying the maximum criteria which is less than 10% (0.1).

$$CR=\frac{CI}{RI}$$ (10)

**Table 3:** Random Index (RI).

*N*	1	2	3	4	5	6	7	8	9
*RI*	0	0	0.52	0.89	1.11	1.25	1.35	1.40	1.45

The value of *CR* ≤ *0*.*1*is acceptable otherwise the pairwise comparison needs to be revised. It means that if the value of CR is less than 0.1, then less than 10% inconsistency is acceptable. Inconsistency is basically the modification required to improve the consistency of the pairwise comparison. But the modification should not be as large as the judgment itself nor so small that it should have no significance, thus it should be of one order smaller scale. The overall inconsistency for a specific comparison should be less than 10% [24]. Eigen Vector (EV_1_) in matrix (12) is obtained through Eq (11), which represents the average value of each row.

$$EV_{i}=\frac{1}{n}\sum_{j=1}^{n}a_{ij}\;\mathrm{where}\;i=1,2,3,\ldots ,n$$ (11)

$$\left[\begin{array}{ccccccc} & N_{1} & N_{2} & N_{3} & N_{4} & N_{5} & EV_{1}\\ N_{1} & 0.074 & 0.148 & 0.083 & 0.037 & 0.047 & 0.078\\ N_{2} & 0.037 & 0.074 & 0.083 & 0.148 & 0.047 & 0.078\\ N_{3} & 0.592 & 0.592 & 0.666 & 0.592 & 0.762 & 0.641\\ N_{4} & 0.148 & 0.037 & 0.083 & 0.074 & 0.047 & 0.078\\ N_{5} & 0.148 & 0.148 & 0.083 & 0.148 & 0.095 & 0.124\\ CR=0.08 & & & & & & \end{array}\right]$$ (12)

The remaining matrices of all pairwise comparisons are solved through the same process. CR against all matrices must be less than 0.1, otherwise recalculate the pairwise comparison. In matrix (13) nodes are compared with respect to *DistNode* (distance from other nodes). The column values of N_1_, N_2_, N_3_, N_4_ and N_5_ are basically the weights obtained after pairwise comparison and E.V is directly calculated by following the same process for *DistCent*.

$$\left[\begin{array}{ccccccc} & N_{1} & N_{2} & N_{3} & N_{4} & N_{5} & EV_{2}\\ N_{1} & 0.10 & 0.18 & 0.09 & 0.06 & 0.17 & 0.119\\ N_{2} & 0.03 & 0.06 & 0.09 & 0.03 & 0.02 & 0.051\\ N_{3} & 0.61 & 0.37 & 0.60 & 0.72 & 0.52 & 0.583\\ N_{4} & 0.20 & 0.18 & 0.09 & 0.12 & 0.17 & 0.156\\ N_{5} & 0.05 & 0.18 & 0.09 & 0.06 & 0.08 & 0.091\\ CR=0.06 & & & & & & \end{array}\right]$$ (13)

MN (Merged Node) criteria is taken in to account for every alternative in matrix (14).

$$\left[\begin{array}{ccccccc} & N_{1} & N_{2} & N_{3} & N_{4} & N_{5} & EV_{3}\\ N_{1} & 1.0 & 0.5 & 0.5 & 0.5 & 2.0 & 0.140\\ N_{2} & 2.0 & 1.0 & 0.5 & 0.5 & 2.0 & 0.185\\ N_{3} & 2.0 & 2.0 & 1.0 & 1.0 & 2.0 & 0.322\\ N_{4} & 2.0 & 2.0 & 0.5 & 0.5 & 2.0 & 0.244\\ N_{5} & 0.5 & 0.5 & 0.5 & 0.5 & 1.0 & 0.106\\ CR=0.04 & & & & & & \end{array}\right]$$ (14)

Nodes are compared with respect to residual energy level (REL) in matrix (15)

$$\left[\begin{array}{ccccccc} & N_{1} & N_{2} & N_{3} & N_{4} & N_{5} & EV_{4}\\ N_{1} & 1.0 & 0.5 & 0.5 & 0.5 & 0.5 & 0.102\\ N_{2} & 2.0 & 1.0 & 2.0 & 0.5 & 0.5 & 0.194\\ N_{3} & 2.0 & 0.5 & 1.0 & 2.0 & 0.5 & 0.194\\ N_{4} & 2.0 & 2.0 & 0.5 & 1.0 & 0.5 & 0.194\\ N_{5} & 2.0 & 2.0 & 2.0 & 2.0 & 1.0 & 0.314\\ CR=0.08 & & & & & & \end{array}\right]$$ (15)

Pairwise comparison of nodes (alternatives) with respect to TCH (Number of times a node has been CH) is shown in matrix (16). All weights of the nodes are same, this is due to the fact that TCH in the first round is same for every node. In the first round every node has TCH = 0 resulting same weights and same E.V.

$$\left[\begin{array}{ccccccc} & N_{1} & N_{2} & N_{3} & N_{4} & N_{5} & EV_{5}\\ N_{1} & 1.0 & 1.0 & 1.0 & 1.0 & 1.0 & 0.2\\ N_{2} & 1.0 & 1.0 & 1.0 & 1.0 & 1.0 & 0.2\\ N_{3} & 1.0 & 1.0 & 1.0 & 1.0 & 1.0 & 0.2\\ N_{4} & 1.0 & 1.0 & 1.0 & 1.0 & 1.0 & 0.2\\ N_{5} & 1.0 & 1.0 & 1.0 & 1.0 & 1.0 & 0.2\\ CR=0.0 & & & & & & \end{array}\right]$$ (16)

#### 4.2.2 Criteria comparison using alternatives (nodes)

The parameters in criteria (DistCent, DistNode, MN, REL and TCH) are compared with respect to all the elements of alternatives (N_1_, N_2_, N_3_, N_4_ and N_5_) one by one and are shown in matrices from (17) to (21) respectively. Matrix (17) shows the pairwise comparison of parameters in accordance to the weights described in Table 2 by keeping in view N1. The column values of *DistCent*, *DistNode*, *MN*, *REL* and *TCH* in matrix (17) are the weights obtained after pairwise comparison and E.V is obtained by following the same process for *DistCent*.

$$\left[\begin{array}{ccccccc} & DistCent & DistNode & MN & REL & TCH & EV_{6}\\ DistCent & 1.0 & 0.25 & 4.0 & 0.16 & 3.0 & 0.113\\ DistNode & 4.0 & 1.0 & 4.0 & 0.2 & 4.0 & 0.217\\ MN & 0.25 & 0.25 & 1.0 & 0.14 & 2.0 & 0.056\\ REL & 6.0 & 5.0 & 7.0 & 1.0 & 9.0 & 0.573\\ TCH & 0.33 & 0.25 & 0.5 & 0.11 & 1.0 & 0.041\\ CR=0.09 & & & & & & \end{array}\right]$$ (17)

$$\left[\begin{array}{ccccccc} & DistCent & DistNode & MN & REL & TCH & EV_{7}\\ DistCent & 1.0 & 0.25 & 2.0 & 0.2 & 3.0 & 0.099\\ DistNode & 4.0 & 1.0 & 4.0 & 0.2 & 6.0 & 0.236\\ MN & 0.5 & 0.25 & 1.0 & 0.14 & 3.0 & 0.071\\ REL & 5.0 & 5.0 & 7.0 & 1.0 & 6.0 & 0.550\\ TCH & 0.33 & 0.16 & 0.33 & 0.16 & 1.0 & 0.042\\ CR=0.09 & & & & & & \end{array}\right]$$ (18)

$$\left[\begin{array}{ccccccc} & DistCent & DistNode & MN & REL & TCH & EV_{8}\\ DistCent & 1.0 & 0.33 & 4.0 & 0.16 & 2.0 & 0.109\\ DistNode & 3.0 & 1.0 & 5.0 & 0.16 & 3.0 & 0.196\\ MN & 0.25 & 0.2 & 1.0 & 0.14 & 0.33 & 0.039\\ REL & 6.0 & 6.0 & 7.0 & 1.0 & 5.0 & 0.573\\ TCH & 0.5 & 0.33 & 3.0 & 0.2 & 1.0 & 0.081\\ CR=0.08 & & & & & & \end{array}\right]$$ (19)

$$\left[\begin{array}{ccccccc} & DistCent & DistNode & MN & REL & TCH & EV_{9}\\ DistCent & 1.0 & 0.25 & 2.0 & 0.16 & 0.5 & 0.068\\ DistNode & 4.0 & 1.0 & 4.0 & 0.16 & 4.0 & 0.213\\ MN & 0.5 & 0.25 & 1.0 & 0.16 & 0.5 & 0.052\\ REL & 6.0 & 6.0 & 6.0 & 1.0 & 6.0 & 0.578\\ TCH & 2.0 & 0.25 & 2.0 & 0.16 & 1.0 & 0.089\\ CR=0.08 & & & & & & \end{array}\right]$$ (20)

$$\left[\begin{array}{ccccccc} & DistCent & DistNode & MN & REL & TCH & EV_{10}\\ DistCent & 1.0 & 0.25 & 3.0 & 0.2 & 3.0 & 0.107\\ DistNode & 4.0 & 1.0 & 5.0 & 0.2 & 4.0 & 0.227\\ MN & 0.33 & 0.2 & 1.0 & 0.12 & 2.0 & 0.055\\ REL & 5.0 & 5.0 & 8.0 & 1.0 & 8.0 & 0.566\\ TCH & 0.33 & 0.25 & 0.5 & 0.12 & 1.0 & 0.044\\ CR=0.07 & & & & & & \end{array}\right]$$ (21)

### 4.3 Unweighted and weighted supermatrix

Table 4 shows the unweighted supermatrix of the ANP model. It contains the local priorities of the nodes obtained from the pairwise comparison matrices (12) to (21). Eigenvectors of each individual matrix are combined, which form the unweighted supermatrix shown in Table 4 (the shaded region represents the corresponding Eigen vectors). This unweighted matrix is then transformed in to weighted supermatrix to make it column stochastic (the sum of each column will be 1) as shown in Table 5. Weighted supermatrix is the outcome of unweighted supermatrix and cluster matrix.

**Table 4:** Unweighted supermatrix.

		Alternatives					Criteria				
		Node1	Node2	Node3	Node4	Node5	DistCent	DistNode	MN	REL	TCH
							(EV_1_)	(EV_2_)	(EV_3_)	(EV_4_)	(EV_5_)
**Alternatives**	**Node1**	0.0000	0.0000	0.0000	0.0000	0.0000	0.0778	0.1189	0.1405	0.1028	0.2000
	**Node2**	0.0000	0.0000	0.0000	0.0000	0.0000	0.0778	0.0511	0.1854	0.1944	0.2000
	**Node3**	0.0000	0.0000	0.0000	0.0000	0.0000	0.6408	0.5833	0.3229	0.1944	0.2000
	**Node4**	0.0000	0.0000	0.0000	0.0000	0.0000	0.0778	0.1562	0.2447	0.1944	0.2000
	**Node5**	0.0000	0.0000	0.0000	0.0000	0.0000	0.1244	0.0905	0.1065	0.3142	0.2000
		**(EV**_**6**_**)**	**(EV**_**7**_**)**	**(EV**_**8**_**)**	**(EV**_**9**_**)**	**(EV**_**10**_**)**	0.0000	0.0000	0.0000	0.0000	0.0000
**Criteria**	**DistCent**	0.1129	0.0993	0.1096	0.0681	0.1072					
	**DistNode**	0.2168	0.2367	0.1968	0.2129	0.2278	0.0000	0.0000	0.0000	0.0000	0.0000
	**MN**	0.0560	0.0712	0.0399	0.0521	0.0550	0.0000	0.0000	0.0000	0.0000	0.0000
	**REL**	0.5727	0.5502	0.5731	0.5780	0.5661	0.0000	0.0000	0.0000	0.0000	0.0000
	**TCH**	0.0416	0.0425	0.0806	0.0890	0.0438	0.0000	0.0000	0.0000	0.0000	0.0000

**Table 5:** Weighted supermatrix.

		Alternatives					Criteria				
		Node1	Node2	Node3	Node4	Node5	DistCent	DistNode	MN	REL	TCH
**Alternatives**	Node1	0.0000	0.0000	0.0000	0.0000	0.0000	0.0389	0.0595	0.0703	0.0514	0.1000
	Node2	0.0000	0.0000	0.0000	0.0000	0.0000	0.0389	0.0256	0.0927	0.0972	0.1000
	Node3	0.0000	0.0000	0.0000	0.0000	0.0000	0.3204	0.2916	0.1614	0.0972	0.1000
	Node4	0.0000	0.0000	0.0000	0.0000	0.0000	0.0389	0.0781	0.1223	0.0972	0.1000
	Node5	0.0000	0.0000	0.0000	0.0000	0.0000	0.0622	0.0453	0.0533	0.1571	0.1000
**Criteria**	DistCent	0.1129	0.0993	0.1096	0.0681	0.1072	0.0000	0.0000	0.0000	0.0000	0.0000
	DistNode	0.2168	0.2367	0.1968	0.2129	0.2278	0.0000	0.0000	0.0000	0.0000	0.0000
	MN	0.0560	0.0712	0.0399	0.0521	0.0550	0.0000	0.0000	0.0000	0.0000	0.0000
	REL	0.5727	0.5502	0.5731	0.5780	0.5661	0.0000	0.0000	0.0000	0.0000	0.0000
	TCH	0.0416	0.0425	0.0806	0.0890	0.0438	0.0000	0.0000	0.0000	0.0000	0.0000

### 4.4 Limit supermatrix

**Fig 6:**
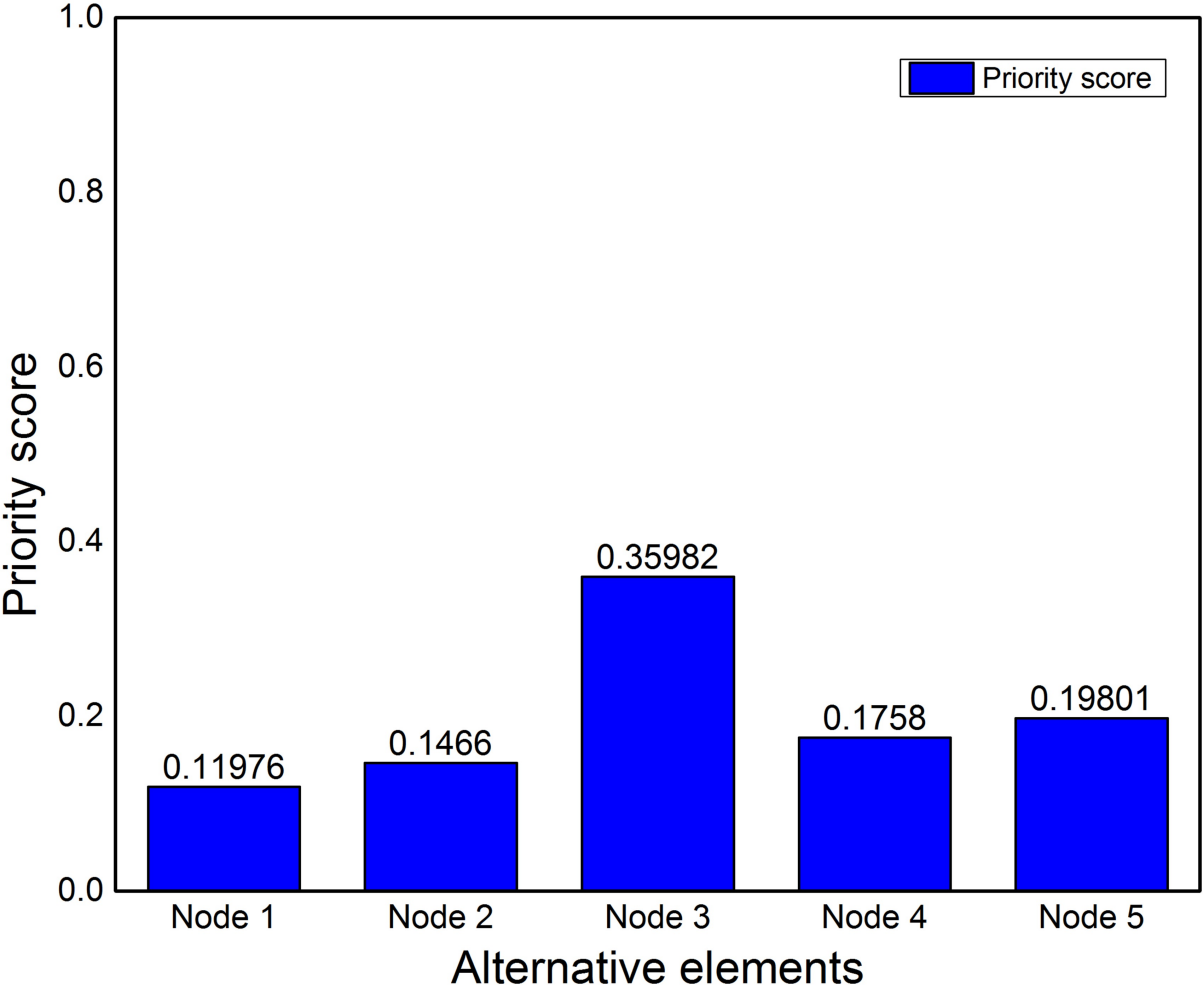
Alternatives priority evaluated applying ANP model.

Limit supermatrix is used to obtain stable weights from weighted supermatrix. All the values in weighted supermatrix are raised to the power of *2k*, where *k* is the random number. Process is repeated until same and stable values are achieved. Limit supermatrix contains the summary of all the pairwise comparison made. It comprises of all indirect impacts between elements. Table 6 shows the limit supermatrix, where we can easily figure out the standing alternative and criteria. It is clear that *Node3* is the most desirable node to be cluster head among these nodes, and *REL* is the most important criteria element among others. Priorities of alternatives are shown in Fig 6 and nodes are ranked accordingly as well. It is clear from Fig 6 that *Node3* is the best choice to be the CH having maximum priority score.

**Table 6:** Limit supermatrix.

		Alternatives					Criteria				
		Node1	Node2	Node3	Node4	Node5	DistCent	DistNode	MN	REL	TCH
**Alternatives**	Node1	0.0299	0.0299	0.0299	0.0299	0.0299	0.0299	0.0299	0.0299	0.0299	0.0299
	Node2	0.0367	0.0367	0.0367	0.0367	0.0367	0.0367	0.0367	0.0367	0.0367	0.0367
	**Node3**	**0.0900**	**0.0900**	**0.0900**	**0.0900**	**0.0900**	**0.0900**	**0.0900**	**0.0900**	**0.0900**	**0.0900**
	Node4	0.0440	0.0440	0.0440	0.0440	0.0440	0.0440	0.0440	0.0440	0.0440	0.0440
	Node5	0.0495	0.0495	0.0495	0.0495	0.0495	0.0495	0.0495	0.0495	0.0495	0.0495
**Criteria**	DistCent	0.0752	0.0752	0.0752	0.0752	0.0752	0.0752	0.0752	0.0752	0.0752	0.0752
	DistNode	0.1035	0.1035	0.1035	0.1035	0.1035	0.1035	0.1035	0.1035	0.1035	0.1035
	MN	0.0629	0.0629	0.0629	0.0629	0.0629	0.0629	0.0629	0.0629	0.0629	0.0629
	REL	0.1923	0.1923	0.1923	0.1923	0.1923	0.1923	0.1923	0.1923	0.1923	0.1923
	TCH	0.0661	0.0661	0.0661	0.0661	0.0661	0.0661	0.0661	0.0661	0.0661	0.0661

## 5. Sensitivity analysis

Sensitivity analysis is highly recommended to check the stability of alternatives ranking. This is used to check the results and ranking of alternatives obtained through ANP model. According to weighted matrix, it should be considered that the elements in alternatives are influenced by the elements in criteria and vice versa. To start with the sensitivity analysis, elements having highest weights are identified first. The impact of these weights must be observed on all other elements (alternatives).

**Fig 7:**
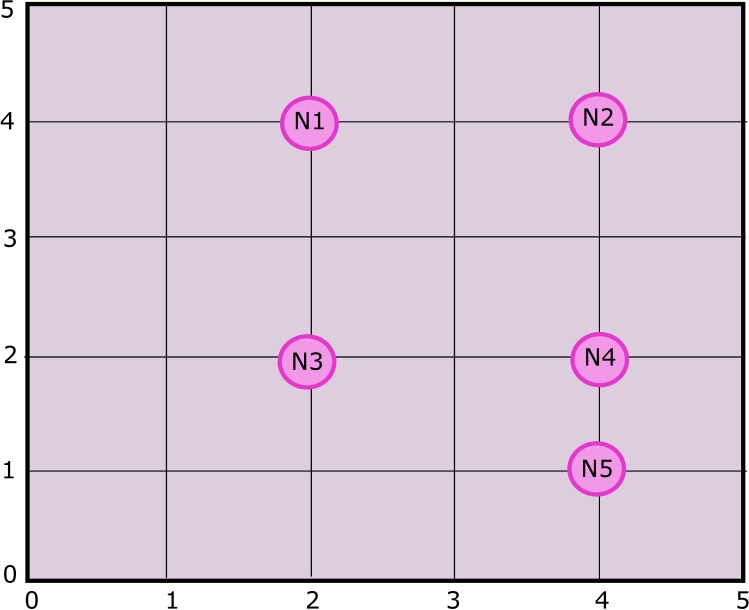
Scenario 1.

**Fig 8:**
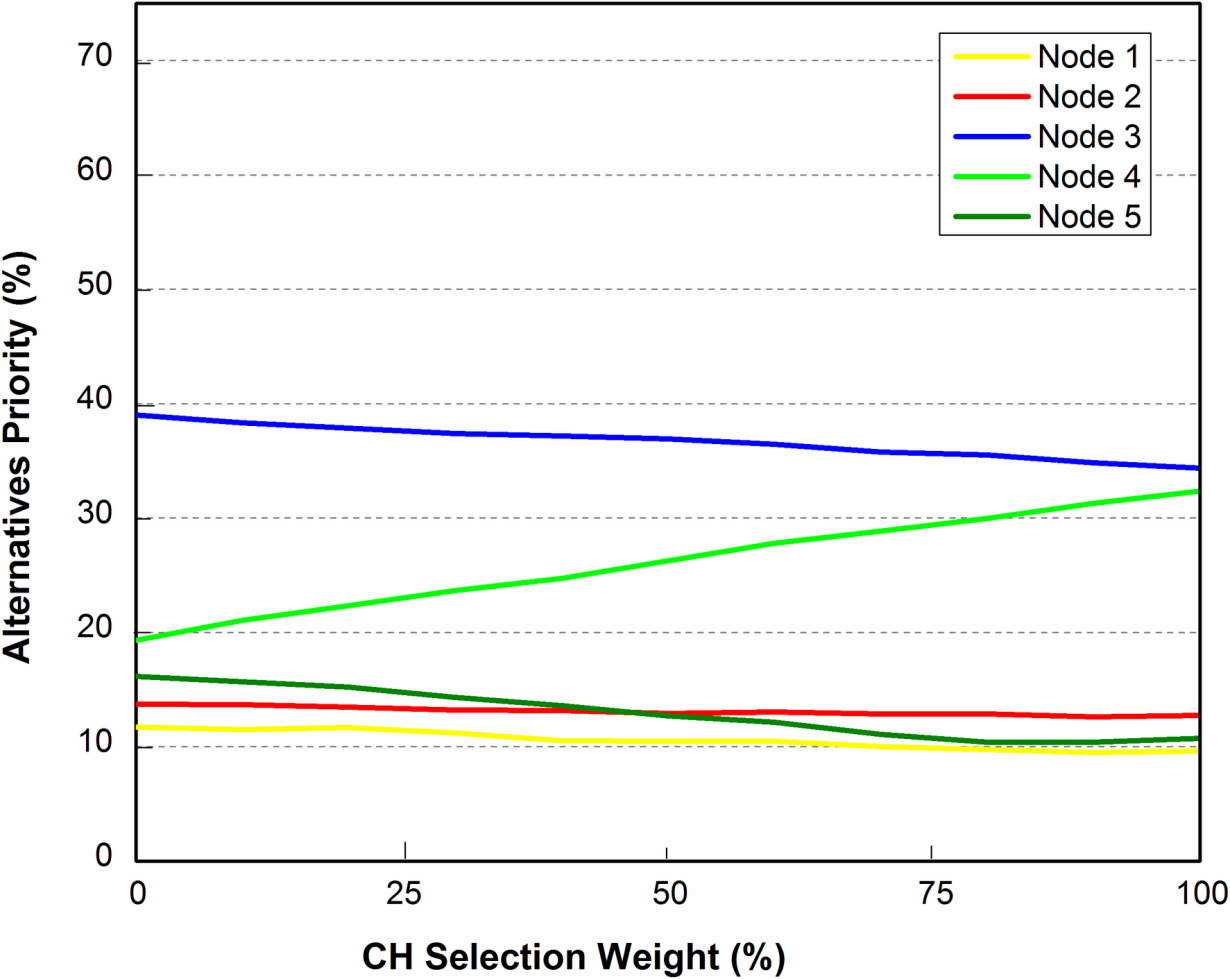
Sensitivity analysis of alternatives with respect to DistNode.

**Fig 9:**
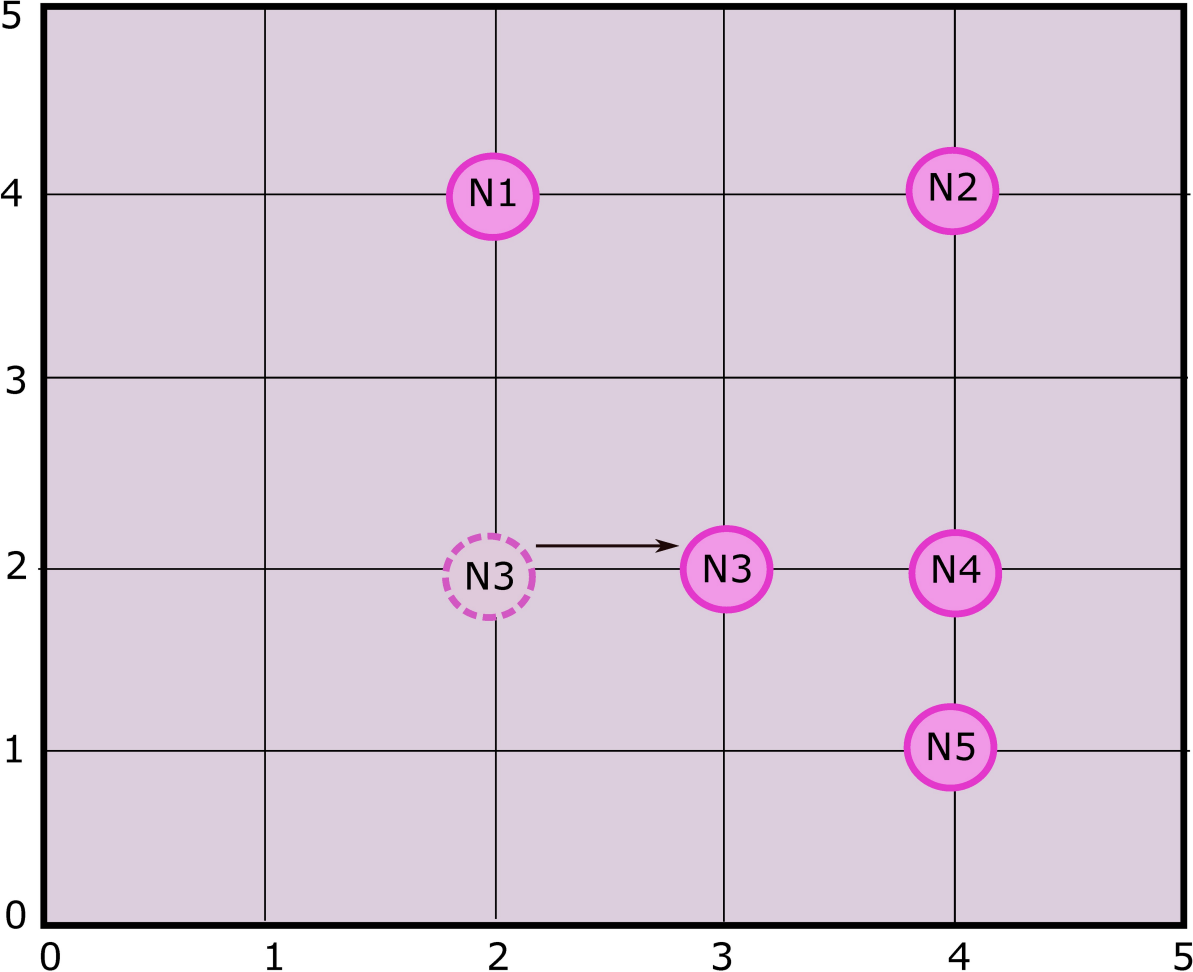
Scenario 2.

**Fig 10:**
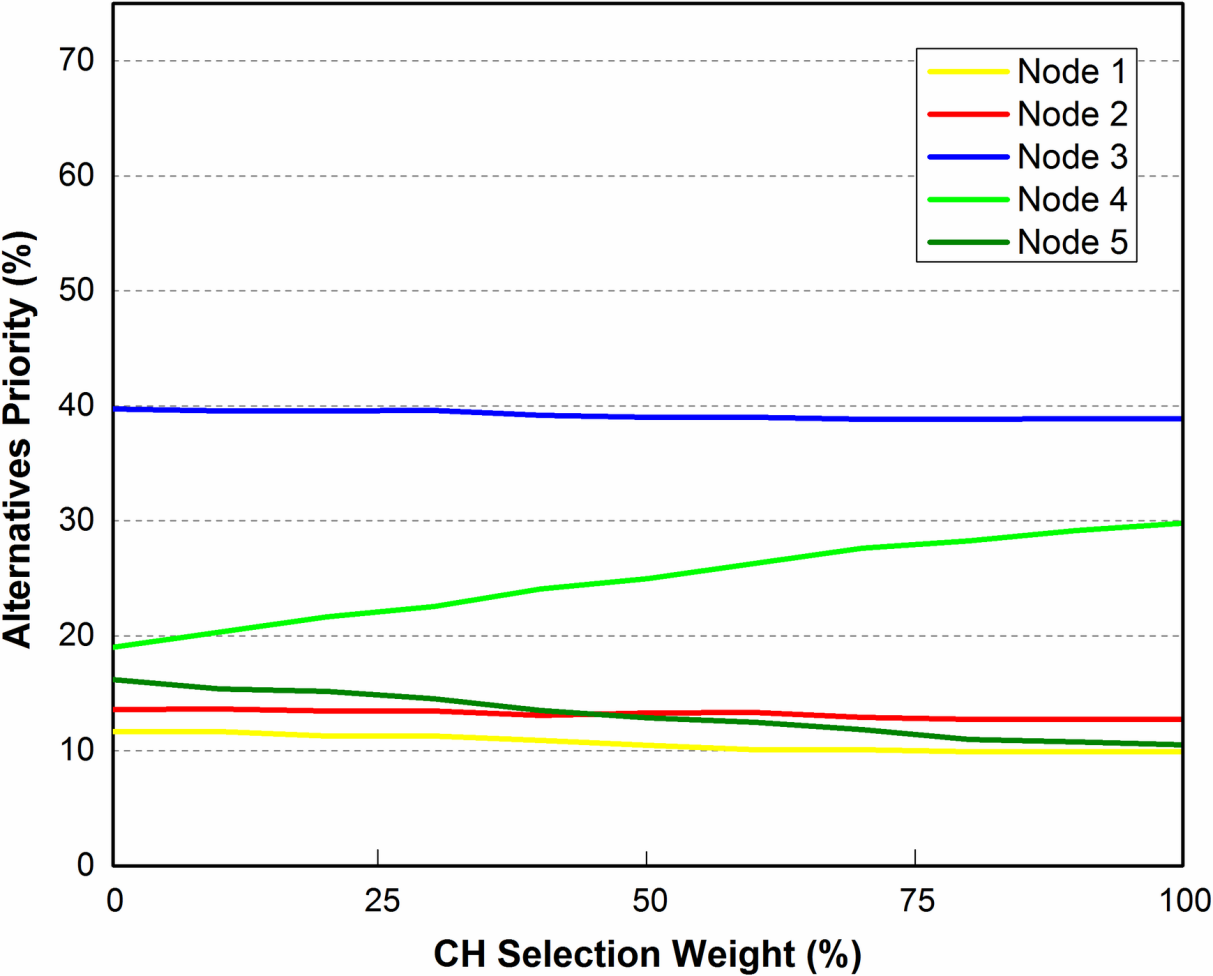
Sensitivity analysis of alternatives with respect to *DistNode*, when N_3_ gets closer to N_4_.

**Fig 11:**
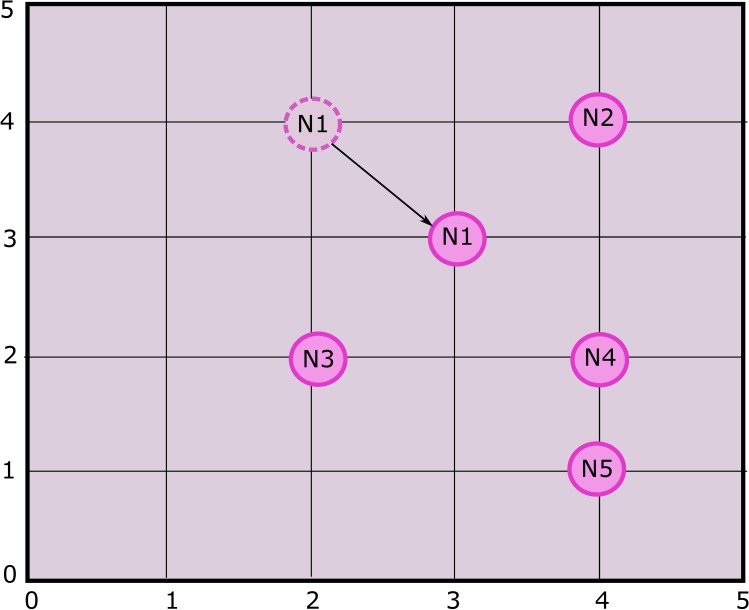
Scenario 3.

**Fig 12:**
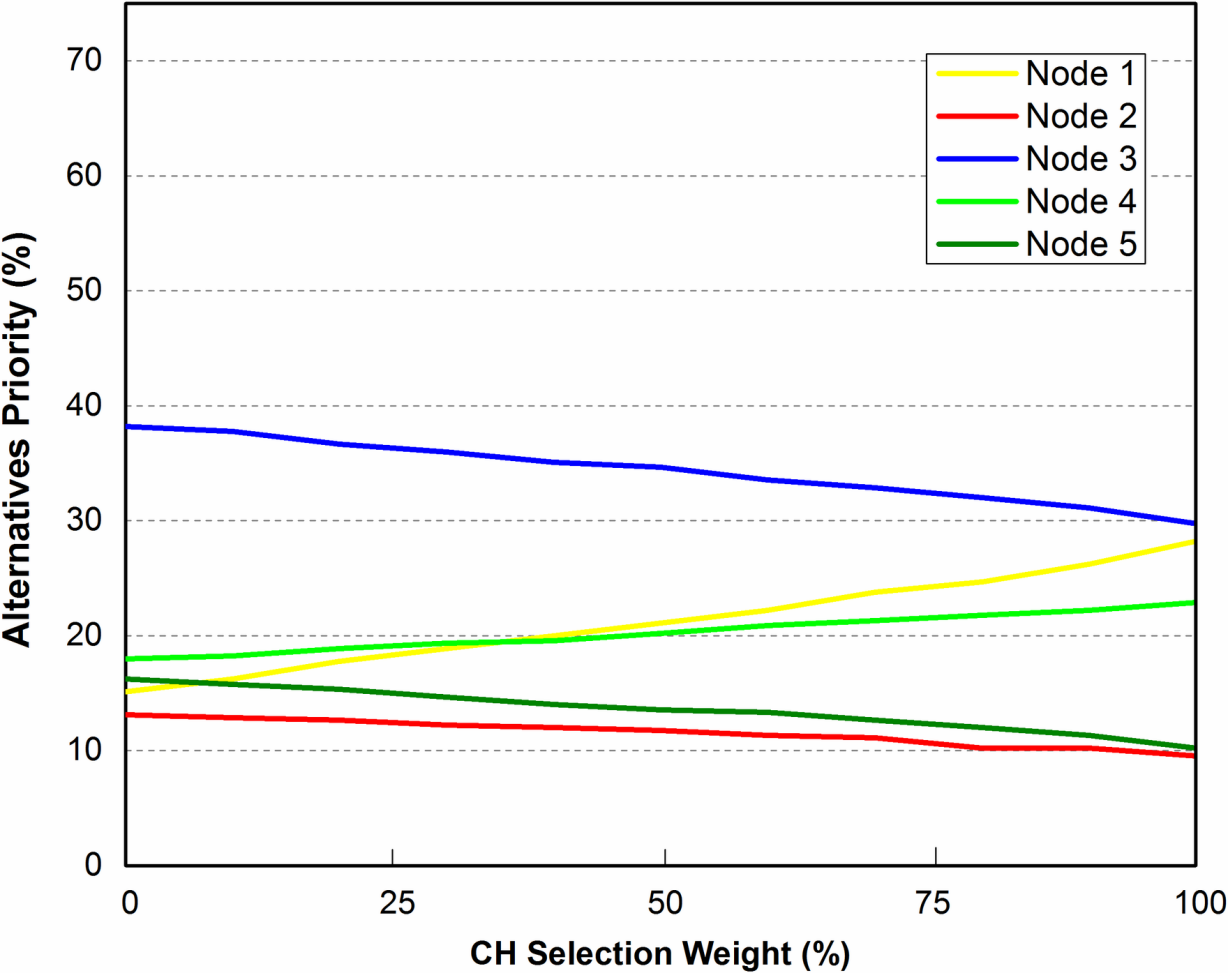
Sensitivity analysis of alternatives with respect to *DistNode*, when N_1_ gets closer to the center of zone.

Referring to Table 6, the highest ranked elements are *REL* and *DistNode* with values of ***0*.*19229*** and ***0*.*10351*** respectively. We have chosen *DistNode*, the second highest ranked element, as initially all nodes of the cluster have the same *REL* value. In this case sensitivity analysis is carried out to check the impact of *DistNode* on the ranking of alternative elements for three different scenarios as shown in Figs 7–12. In these figures, the x-axis represents cluster head selection weight (goal) and y-axis represents alternatives priority (nodes).

Initially we applied this process to only one cluster containing 5 nodes (N_1_, N_2_, N_3_, N_4_ and N_5_ as shown in Fig 1 where Zone/Cluster 6 is considered) against REL, DistNode, DistCent, TCH and MN one by one respectively. The same process is extended to all other clusters in a network containing different number of nodes, where the criteria parameters remain same while alternatives vary according to the number of nodes in a specific cluster.

### 5.1 Scenario 1

The basic configuration of 5 sensor nodes (represented by N_1_, N_2_, …, N_5_) each one at a specified location, generally denoted by *(x*_*i*_, *y*_*i*_*)* where *i* = 1, 2, 3, 4, 5 as shown in Fig 7. In sensitivity analysis, *DistNode* parameter is considered in existing topology in order to check the corresponding ranking of the nodes. The result shown in Fig 8, reveal that node 3 is of highest rank and therefore the most suitable candidate for CH selection.

### 5.2 Scenario 2

Sensitivity analysis aims at checking the stability of the rankings of alternatives. In scenario 2 as shown in Fig 9, the position of N_3_ is slightly changed in direction of N_4_ i.e. from location (2, 2) to new location (2, 3). Changing the location of the N_3_ requires re-computation of the pairwise comparisons of alternatives (nodes). The sensitivity analysis obtained from the recomputed pairwise comparison show that N_3_ is still the most suitable node to be selected for CH as shown in Fig 10. It is concluded that the alternatives are stable and robust as ranking of the alternatives didn’t change by slightly changing the criteria parameter, in this case *DistNode*.

### 5.3 Scenario 3

To check the stability and robustness of the alternatives ranking even more, we targeted N_1_ to vary its position as shown in Fig 11. N_1_ was moved closer to the center of grid. While considering distance from other nodes (*DistNode*), N_1_ seems to be the good choice. Sensitivity analysis is shown in Fig 12. It is clear that N_3_ is still the high ranked node but there is a notable change in the ranks of alternatives i.e. N_1_ and N_4_. It is due to the fact that N_1_ has more neighbor nodes than it had in scenario 1 and 2, thus making it second highest priority node to be selected for CH.

The whole process of ANP is applied on the entire network for all zones containing different number of nodes. The BS will maintain alternatives priority list (APL) for each individual zone containing weights of nodes. In a particular zone the node with maximum weight is selected as CH. The BS then share the APL information with the newly selected CH. The CH advertise itself for member nodes to send their sensed data to it which is to be directed to the BS eventually.

## 6. Cluster head reselection procedure

In the proposed method, role of CH is rotated to minimize energy consumption and to avoid early depletion of node. Instead of periodic reselection of CH that leads to network overhead and high energy consumption, the proposed method initiates the process when required. Moreover, the reselection process is zone dependent thus the entire network is not disturbed in the reselection process. The reselection process is initiated based on experimentally obtained optimum threshold value (TV) [17]. If energy level of a CH is less than TV then the process of reselection will be initiated as shown in Algorithm 1. The reselection score (ReSS) is calculated based on three distinct weighted parameters such as Node_weight (fetched from APL), Node_EL_ (energy level of a node) and Pr_TCH_ (Priority of a node to be CH) as shown in Eq (22). The Pr_TCH_ is basically the number of times a node has been CH with it priority. Moreover, TCH = 0 means the node has not been cluster head and will have high priority. Furthermore, a high score of TCH will decrease the priority as shown in Eq (23). The weights w_1_, w_2_ and w_3_ are assigned to Node_weight, Node_EL_ and Pr_TCH_ respectively as shown in Eq (22). We recommend weights to be assigned as w_2_ > w_1_ > w_3_ and w_1_ + w_2_ + w_3_ = 1. This criteria for parameter selection is decided after empirical evaluation. It assigns highest weight to energy level of the node as it is the most important factor. The second important being the weight assigned to it by ANP and third important weight is assigned that indicates the frequency of being selected as CH.

ReSS=(w1×Node_weight)+(w2×NodeEL)+(w3×PrTCH) (22)

$${\mathrm{Pr}}_{TCH}=\frac{1}{\left(1+TCH\right)}$$ (23)

**Algorithm 1. Cluster head reselection procedure**.

**Procedure:** Cluster head reselection

**Input:** Energy level of nodes in a cluster, Node_EL_; Alternatives Priority List of individual cluster, APL_CID; Number of times a node has been CH, TCH.

**If** (CH_EL_ ≤ TV) **then**

      APL_CID = ANP(ClusterID)

      **For** n = 1 to NumberOfNodesInCluster

            Pr_TCH_ = 1/(1+TCH(n))

            ReSS(n) = w1*Node_weight(n) + w2*Node_EL_(n) + w3*Pr_TCH_

      **Next**

      CHead = IndexOf(Max(ReSS))

            Advertise CHead

            Member_Nodes join CHead

**Else**

      Remain CHead

End procedure

## 7. Results and discussions

**Fig 13:**
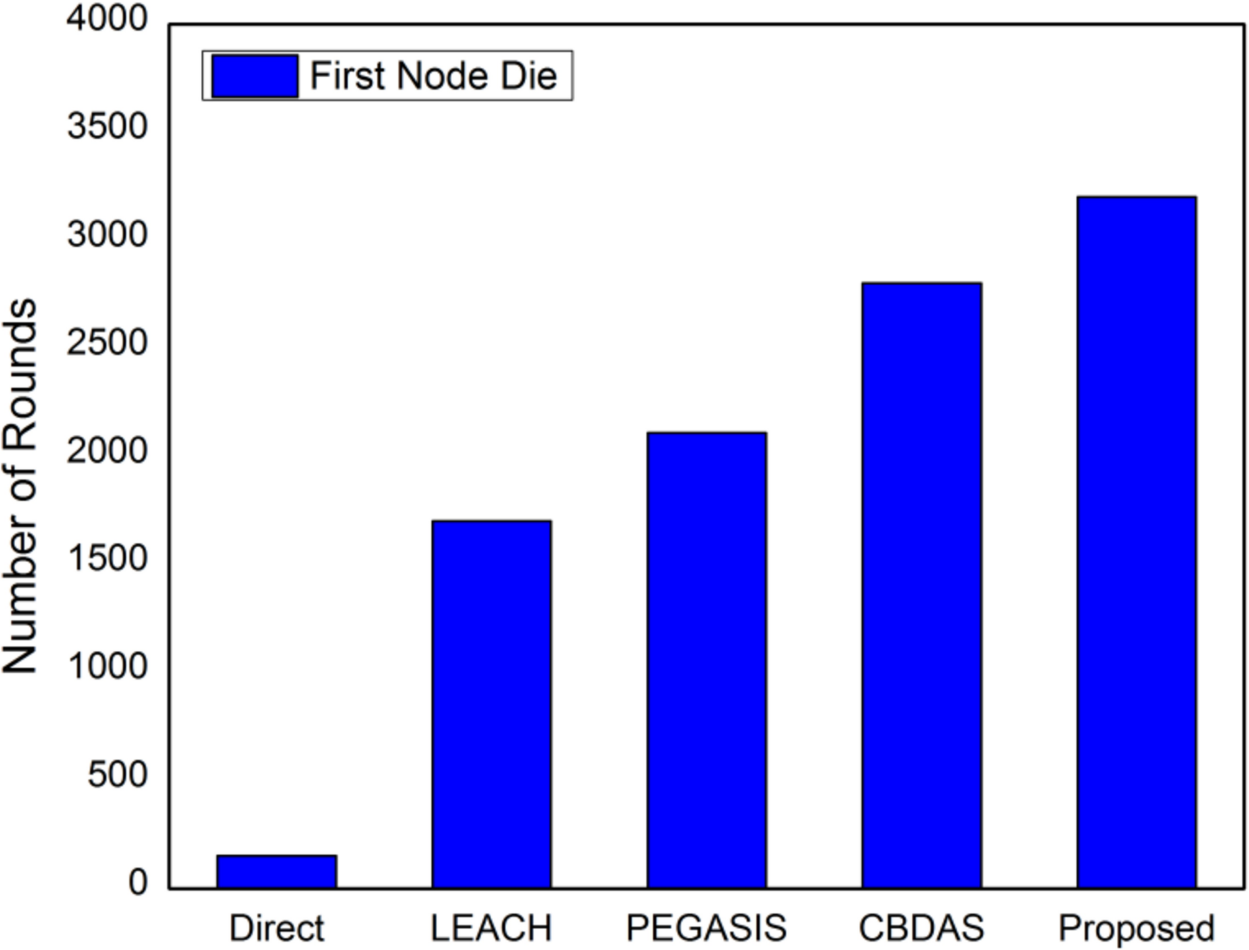
Number of rounds when first node dies.

**Fig 14:**
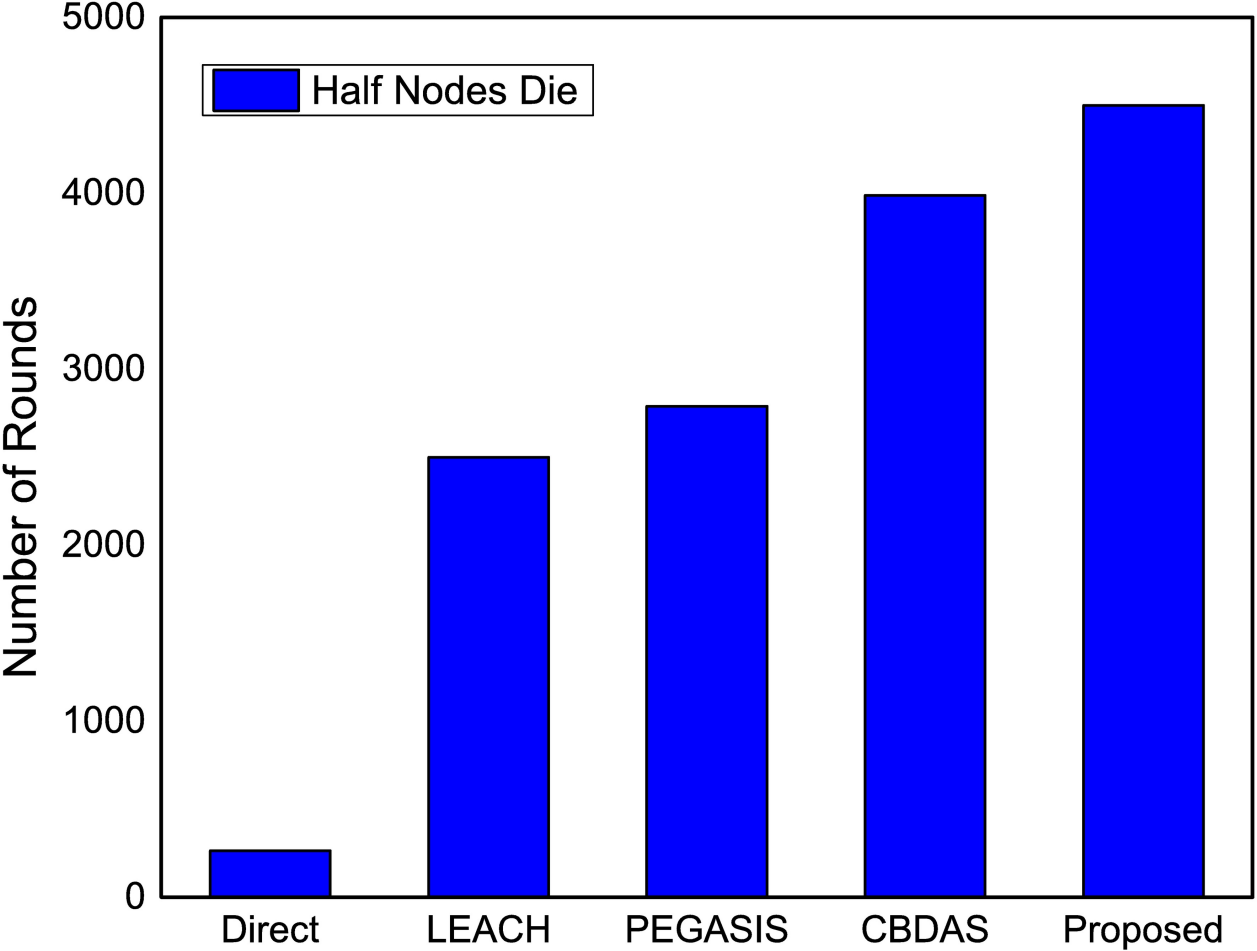
Number of rounds when half nodes die in the network.

**Fig 15:**
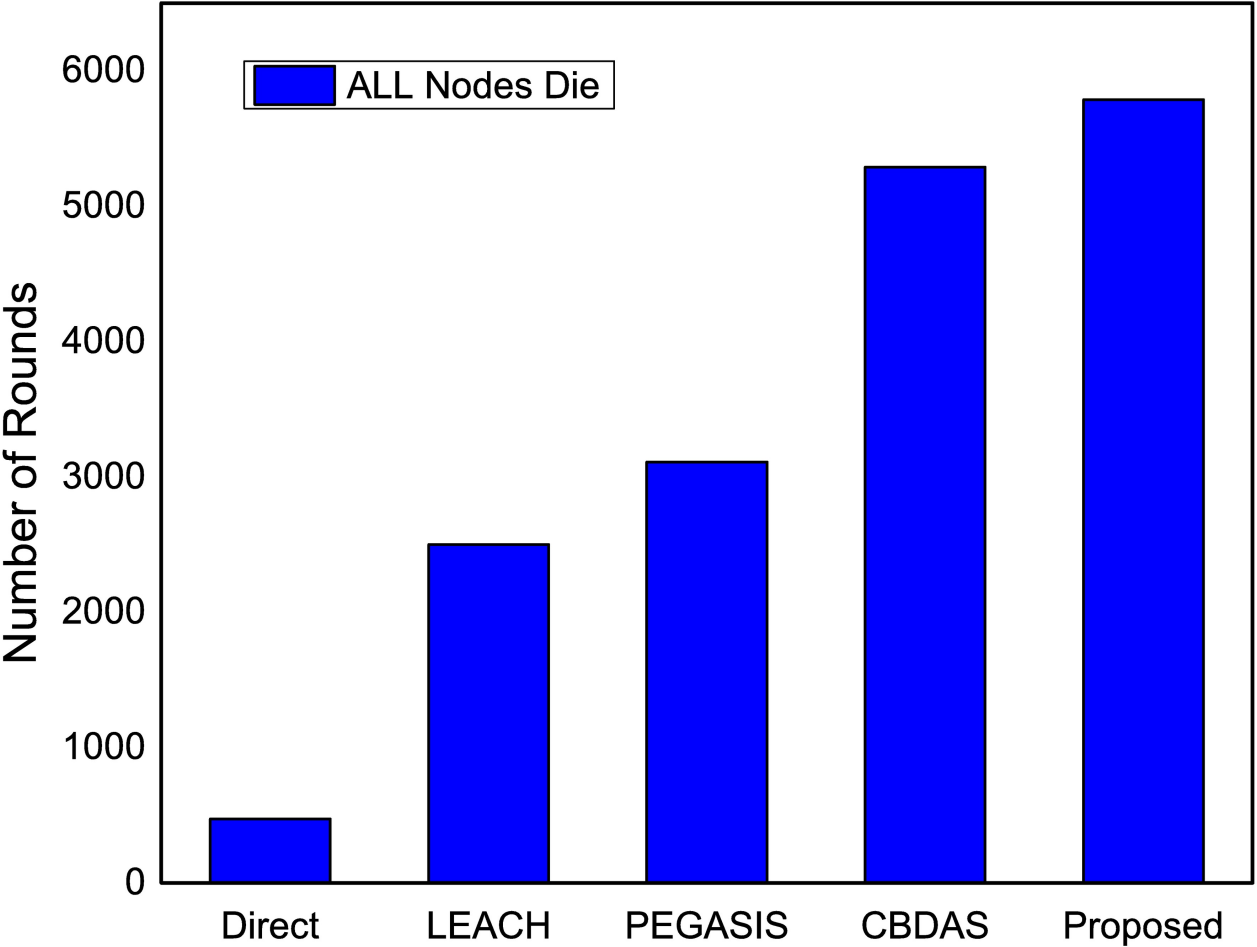
Number of rounds when all nodes dies in the network.

The performance of the proposed method using ANP approach is compared with existing state-of-the art energy efficient protocols. The proposed approach is evaluated in terms of network lifetime using first node die (FND), half nodes die (HND) and all nodes die (AND). In the proposed method, a CH is selected based on ANP approach. After certain number of rounds the CH is rotated to preserve the energy, prolonging network stability, and to avoid network partitioning which typically results in unreachable segments. If energy level of a CH decreases below the defined threshold the reselection of CH is initiated for a specific zone. The CH selection based on the parameters mentioned in sub section 4.1 and the reselection process particular to the zone improves energy consumption and subsequently prolongs the network lifetime as shown in Figs 13–15.

### 7.1 Simulation setup

The total number of sensor nodes (S*i*) deployed are N, i ϵ {1, 2, 3,…, N} in a squared targeted area. The entire area is divided in to virtual grids of M x M dimension, for example M = 4 means that total number of grids are 16. The parameters used in simulation are presented in Table 7. The energy consumption of all nodes is evaluated using first order radio model [5]. In the proposed work, few assumptions are made regarding sensor nodes and deployment area which are:

- The position of BS is outside the deployment area and is known to all nodes.
- The BS and all sensor nodes are static (don’t change their position) once deployed.
- Sensor nodes are deployed in homogenous environment having same initial energy E°.
- All the sensor nodes already have the information of energy and location (coordinates).

**Table 7:** Simulation parameters.

Parameters	Value
Network Area (meters)	100 x 100
Number of nodes (N)	300
Initial Energy (Joules)	1.0
Packet size (bits)	2000
Number of Grids	16
E_amp_ (pJ / bit / m^2^)	100
E_elec_ (nJ / bit)	50
E_DA_ (nJ / bit / signal)	5
W_1_	0.3
W_2_	0.5
W_3_	0.2

### 7.2 Performance evaluation of the proposed method

The proposed method is compared with the related existing energy efficient protocols in terms of network lifetime by considering first node die, half nodes and all nodes die in a network. The results show that the proposed method outperforms the existing protocols in terms of maximizing the overall network lifespan.

In Fig 13, number of rounds are calculated when the first node dies in a network. The proposed method show better results in terms of network stability when first node dies as compared to LEACH, PEGASIS, CBDAS, and Direct as shown in Fig 13. In the proposed method, CH selection is based on five distant parameters (REL, DistCent, DistNodes, TCH and MN) that leads to more efficient selection of node as CH resulting in more stable network lifetime. In direct approach the nodes directly sends data to BS resulting in early depletion of energy. Although LEACH performs far better than direct but as the CH selection is random and might select a node that has low residual energy, therefore, results in less number of rounds as compared to PEGASIS, CBDAS and proposed method.

The proposed method is analyzed when half of the nodes die taking in to account the number of rounds. As in Fig 14 the proposed method maximizes network lifetime by approximately 79.9%, 61.3% and 12.8% in comparison with LEACH, PEGASIS and CBDAS respectively. The ANP based selection improves the selection process and results in optimum CH selection that minimizes the reselection process which in turns prolongs network lifetime. In Fig 15, the proposed method alleviates number of rounds in terms of all nodes die. The proposed method maximizes number of rounds by approximately 131.4%, 86.14% and 9.4% in comparison with LEACH, PEGASIS and CBDAS, respectively.

The overall results conclude that the proposed method selects the most optimal and efficient CH as compared to LEACH with random selection of CH by not considering residual energy. The PEGASIS and CBDAS outperforms LEACH as they results in optimum CH. However, the proposed technique minimizes energy consumption by selecting optimum CH based on distinct parameters. Moreover, it reduces reselection process and initiated when required in a zone results in better energy consumption.

## 8. Conclusion and future work

This paper has attempted to solve CH selection problem in WSN by using ANP which is a multi-criteria decision analysis tool. CH selection in WSN involves tuning of several inter-related parameters such as residual energy level, distance from center of zone and others, which were taken as criteria parameters for the ANP process. Mathematical framework was provided for applying ANP model on CH selection. The mathematical framework was then tested for three different scenarios. In first scenario, a cluster of 5 nodes was considered for ANP based cluster head selection. The criteria parameters; REL, DistCent, DistNodes, TCH and MN were taken into account. Limit matrix (Table 6) shows that REL parameter has the highest value 0.1923. As initially REL is same for all nodes, therefore the second highest parameter (DistNodes with value 0.1035) is selected for sensitivity analysis. Node 3 was evaluated to be the best node for CH selection with priority value of approximately 40%. For second scenario, Node 3 was moved a bit closer to Node 4 and 5 and sensitivity analysis was checked. The result showed that Node 3 is still the best node to be the CH. In scenario 3 in order to check the stability of alternatives ranking, Node 1 was moved to the center of the zone. Sensitivity analysis showed that node 3 was still the best choice for CH selection.

In all cases, results illustrated that alternatives ranking was stable and in all three scenarios Node 3 was selected to be the best alternative node for cluster head. Here we can conclude that ANP can be used as one of the alternative method for cluster head selection in wireless sensor network. The limit matrix represents selection priority for all the candidate nodes where the node with the highest priority weight is selected as CH. In addition to this, the limit matrix also reports priority weights for all criteria parameters, which can be used to optimize the criteria list for efficient cluster head selection by eliminating selection parameters with the low weights thus minimizing computational complexity of the ANP process. Additionally this information can be used for optimization of parameters and could serve as input to other cluster head selection techniques. Finally, the proposed system is applied to entire network and compared with existing related clustering protocols. The results show that the proposed method using ANP approach extends network lifespan as compared to direct, LEACH, PEGASIS and CBDAS. In future, this work can be used with other existing clustering approaches in context of different sizes of a network. The ANP method can be used for optimization of criteria parameters through limit matrix, where the least important criteria can be identified.

## References

[1] Batra PK, Kant K. LEACH-MAC: a new cluster head selection algorithm for Wireless Sensor Networks. Wireless Networks. 2016;22(1):49–60.

[2] Hacioglu G, Kand VFA, Sesli E. Multi objective clustering for wireless sensor networks. Expert Systems with Applications. 2016;59:86–100.

[3] Aslam N, Phillips W, Robertson W, Sivakumar S. A multi-criterion optimization technique for energy efficient cluster formation in wireless sensor networks. Information Fusion. 2011;12(3):202–12.

[4] Saaty TL. What is the analytic hierarchy process? Mathematical models for decision support: Springer; 1988 p. 109–21.

[5] Heinzelman WR, Chandrakasan A, Balakrishnan H, editors. Energy-efficient communication protocol for wireless microsensor networks. System sciences, 2000 Proceedings of the 33rd annual Hawaii international conference on; 2000: IEEE.

[6] Wang Q, Xu K, Hassanein H, Takahara G, editors. Swatch: A stepwise adaptive clustering hierarchy in wireless sensor networks. International Conference on Research in Networking; 2005: Springer.

[7] Handy M, Haase M, Timmermann D, editors. Low energy adaptive clustering hierarchy with deterministic cluster-head selection. Mobile and Wireless Communications Network, 2002 4th International Workshop on; 2002: IEEE.

[8] Ali MS, Dey T, Biswas R, editors. ALEACH: Advanced LEACH routing protocol for wireless microsensor networks. Electrical and Computer Engineering, 2008 ICECE 2008 International Conference on; 2008: IEEE.

[9] Ye M, Li C, Chen G, Wu J, editors. EECS: an energy efficient clustering scheme in wireless sensor networks. PCCC 2005 24th IEEE International Performance, Computing, and Communications Conference, 2005; 2005: IEEE.

[10] Bsoul M, Al-Khasawneh A, Abdallah AE, Abdallah EE, Obeidat I. An energy-efficient threshold-based clustering protocol for wireless sensor networks. Wireless personal communications. 2013;70(1):99–112.

[11] Younis O, Fahmy S. HEED: a hybrid, energy-efficient, distributed clustering approach for ad hoc sensor networks. IEEE Transactions on mobile computing. 2004;3(4):366–79.

[12] Lindsey S, Raghavendra CS, editors. PEGASIS: Power-efficient gathering in sensor information systems. Aerospace conference proceedings, 2002 IEEE; 2002: IEEE.

[13] Yu Y, Song Y, editors. An energy-efficient chain-based routing protocol in wireless sensor network. 2010 International Conference on Computer Application and System Modeling (ICCASM 2010); 2010: IEEE.

[14] Mahajan S, Mahotra J. A novel chain based wireless data sensor network (ECBSN) technique. Int J Comput Sci Telecommun. 2011;2:83–7.

[15] Sharma T, Joshi R, Misra M, editors. GBDD: grid based data dissemination in wireless sensor networks. Advanced Computing and Communications, 2008 ADCOM 2008 16th International Conference on; 2008: IEEE.

[16] Chiang Y-K, Wang N-C, Hsieh C-H, Chiang Y-K, Wang N-C, Hsieh C-H. A cycle-based data aggregation scheme for grid-based wireless sensor networks. Sensors 2014: 8447–8464. doi: 10.3390/s140508447 24828579 10.3390/s140508447 PMC4063037

[17] Farman H, Javed H, Ahmad J, Jan B, Zeeshan M. Grid-based Hybrid Network Deployment Approach for Energy Efficient Wireless Sensor Networks. Journal of Sensors. 2016;2016:14.

[18] Ishizaka A, Nemery P. Multi-criteria decision analysis: methods and software: John Wiley & Sons; 2013.

[19] Mendoza GA, Martins H. Multi-criteria decision analysis in natural resource management: A critical review of methods and new modelling paradigms. Forest Ecology and Management. 2006;230(1–3):1–22.

[20] Büyüközkan G, Kahraman C, Ruan D. A fuzzy multi-criteria decision approach for software development strategy selection. International Journal of General Systems. 2004;33(2–3):259–80.

[21] Chengyi Xia LW, Shiwen Sun, Juan Wang. An SIR model with infection delay and propagation vector in complex networks. Nonlinear Dynamics. 2012;69(3):927–34.

[22] Yin Y, Shi J, Li Y, Zhang P, editors. Cluster head selection using analytical hierarchy process for wireless sensor networks. 2006 IEEE 17th International Symposium on Personal, Indoor and Mobile Radio Communications; 2006: IEEE.

[23] Molinos-Senante M, Gómez T, Caballero R, Hernández-Sancho F, Sala-Garrido R. Assessment of wastewater treatment alternatives for small communities: An analytic network process approach. Science of The Total Environment. 2015;532:676–87. doi: 10.1016/j.scitotenv.2015.06.059 26119382 10.1016/j.scitotenv.2015.06.059

[24] Boateng P, Chen Z, Ogunlana SO. An Analytical Network Process model for risks prioritisation in megaprojects. International Journal of Project Management. 2015;33(8):1795–811.

[25] Nazir S, Anwar S, Khan SA, Shahzad S, Ali M, Amin R, et al, editors. Software component selection based on quality criteria using the analytic network process Abstract and Applied Analysis; 2014: Hindawi Publishing Corporation.

[26] Sun S, Wu Y, Ma Y, Wang L, Gao Z, Xia C. Impact of Degree Heterogeneity on Attack Vulnerability of Interdependent Networks. Scientific Reports. 2016;6. 10.1038/srep32983 PMC5016735 27609483

[27] Saaty TL. Decision making with dependence and feedback: The analytic network process. Pittsburgh. RWS Publications. 2001;7:557–70.

[28] Saaty TL. Theory and applications of the analytic network process: decision making with benefits, opportunities, costs, and risks: RWS publications; 2005.

[29] Saaty TL. How to make a decision: the analytic hierarchy process. European journal of operational research. 1990;48(1):9–26. 10.1016/0377-2217(90)90060-o 11659401

[30] Ishizaka A, Labib A. Analytic hierarchy process and expert choice: Benefits and limitations. Or Insight. 2009;22(4):201–20.

